# Is there any evidence for milder courses of monkeypox virus infections with childhood smallpox vaccination?

**DOI:** 10.1007/s15010-022-01923-7

**Published:** 2022-09-20

**Authors:** Christian Hoffmann, Eva Wolf, Heiko Jessen, Markus Bickel, Christoph Boesecke, for the DAIG and DAGNAE

**Affiliations:** 1 Infektionsmedizinisches Centrum Hamburg Stadtmitte, Glockengiesserwall 1, 20095 Hamburg, Germany; 2 University Hospital of Schleswig-Holstein, Campus Kiel, Germany; 3 MUC Research GmbH, Munich, Germany; 4 Praxis Jessen2 + Kollegen, Berlin, Germany; 5 Infektio Research GmbH & Ko KG, Frankfurt, Germany; 6 Department of Medicine I, University Hospital of Bonn, Bonn, Germany; 7 German Centre for Infection Research (DZIF), Partner-Site Cologne-Bonn, Bonn, Germany

**Keywords:** Monkeypox, MPXV infection, Smallpox vaccination

Since May 2022, unusually high numbers of human monkeypox virus (MPXV) infections are being reported across Europe and other regions [1]. On July 23, 2022, WHO declared that the current outbreak represents a public health emergency of international concern and recommended targeted immunization for persons at high risk of exposure for MPXV [2]. Laboratory and animal studies, as well as epidemiological observations mainly from Africa suggest that smallpox vaccination may provide some protection against MPXV [3]. However, in a small outbreak in the US in 2003, no significant differences in serious clinical conditions or complications were seen between vaccinated and unvaccinated individuals [4]. In the absence of controlled clinical trials, we were interested if smallpox vaccination could alter the clinical picture of MPXV infection.

**Fig. 1:**
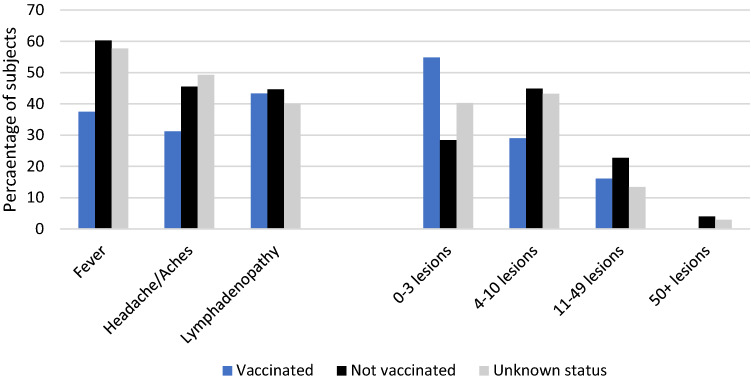
Percentages of subjects with systemic symptoms and number of lesions, according to documented smallpox vaccination status (*n* = 298)

In our ongoing nationwide observational study of 546 MPVX infections among men who have sex with men (MSM), the acute clinical presentation did not differ between MSM with and without HIV infection [5]. For the present analysis, 298 MPVX patients with at least 7 days of follow-up were grouped according to documented smallpox vaccine during childhood (33 vaccinated, 191 unvaccinated, and 74 with unknown status). Hospitalization rates due to MPXV infection were 9·1% in vaccinated persons and 6·8% in unvaccinated persons. However, acute systemic symptoms such as fever, headache or body aches were lower in vaccinated persons, while rates of limited cutaneous disease (defined as 0–3 lesions) were higher (Fig. 1). In a logistic regression analysis including HIV status, PrEP use and vaccination status, odds ratio of limited disease without systemic symptoms was 4·17 with vaccination (95% confidence interval 1·63–10·65, *p* = 0·003).

Our results may provide some limited evidence for a milder course of disease with a history of a smallpox vaccination. However, hospitalization rates were not lower in vaccinated persons. As hospitalizations probably correlate not only with vaccination history but also with the nature and route of exposure in the current outbreak as well as age and comorbidities, it may be challenging to demonstrate any clinical benefit of vaccines even in large populations, based on hospitalization rates alone. We believe that an accurate, consensual index of MPXV infection severity and complications is imperative. Such an index, preferably considering systemic symptoms as well as lesion numbers, could not only help to demonstrate clinical benefits of the current smallpox vaccination campaign, but also to evaluate potential risk factors for disease severity and the efficacy of future MPXV therapies.
